# Retrospective Study Looking at Cinacalcet in the Management of Hyperparathyroidism after Kidney Transplantation

**DOI:** 10.1155/2017/8720283

**Published:** 2017-03-13

**Authors:** Habib Mawad, Hugues Bouchard, Duy Tran, Denis Ouimet, Jean-Philippe Lafrance, Robert Zoël Bell, Sarah Bezzaoucha, Anne Boucher, Suzon Collette, Vincent Pichette, Lynne Senécal, Michel Vallée

**Affiliations:** Division of Nephrology, Maisonneuve-Rosemont Hospital and the Department of Medicine, University of Montreal, Montreal, QC, Canada

## Abstract

*Objectives.* The primary objective of this study is to evaluate the use of cinacalcet in the management of hyperparathyroidism in kidney transplant recipients. The secondary objective is to identify baseline factors that predict cinacalcet use after transplantation.* Methods.* In this single-center retrospective study, we conducted a chart review of all patients having been transplanted from 2003 to 2012 and having received cinacalcet up to kidney transplantation and/or thereafter.* Results.* Twenty-seven patients were included with a mean follow-up of 2.9 ± 2.4 years. Twenty-one were already taking cinacalcet at the time of transplantation. Cinacalcet was stopped within the first month in 12 of these patients of which 7 had to restart therapy. The main reason for restarting cinacalcet was hypercalcemia. Length of treatment was 23 ± 26 months. There were only 3 cases of mild hypocalcemia. There was no statistically significant association between baseline factors and cinacalcet status a year later.* Conclusions.* Discontinuing cinacalcet within the first month of kidney transplantation often leads to hypercalcemia. Cinacalcet appears to be an effective treatment of hypercalcemic hyperparathyroidism in kidney transplant recipients. Further studies are needed to evaluate safety and long-term benefits.

## 1. Introduction

Successful kidney transplantation corrects many abnormalities of bone and mineral metabolism associated with end stage renal disease (ESRD). Nevertheless, hyperparathyroidism does not completely resolve in a significant number of patients even after several years of restored renal function [1]. The severity of preexisting hyperparathyroidism seems to be one of the most important predictors of persistence after transplantation [2]. Other factors include time spent on dialysis and longstanding hyperparathyroidism. The ongoing functional demand for parathyroid hormone (PTH) in ESRD causes morphological transformation of parathyroid tissue [3]. Following kidney transplantation, PTH levels usually decrease considerably within the first 3 to 6 months [4]. However, complete normalization does not often ensue because of the slow involution rate of hyperplastic tissue [5].

Residual hyperparathyroidism in kidney transplant recipients is commonly associated with hypercalcemia which generally manifests 3 to 6 months after transplantation [6]. Persistent hyperparathyroidism has been identified as an independent risk factor for fractures in kidney transplant recipients [7]. Furthermore, It has been associated with decreased allograft survival and increased mortality [8]. Optimal management of hypercalcemic hyperparathyroidism in kidney transplant recipients is not known. Many transplant nephrologists favor parathyroidectomy as primary approach. Observational studies have suggested that cinacalcet might also be an effective option [9]. In a recent randomized controlled trial, cinacalcet was found to be superior to placebo in correcting hypercalcemia and PTH levels [10]. There were no new safety signals although follow-up was rather short. These findings are very encouraging but further studies are needed particularly in regard to long-term clinical outcomes [11]. Moreover, there is an unresolved issue as to whether cinacalcet should be discontinued or pursued at the time of kidney transplantation.

The primary objective of this study is to evaluate the patterns of use of cinacalcet in the management of hyperparathyroidism in a transplant setting. We will report our local experience and compare the different management options that were attempted. The secondary objective is to identify baseline factors that predict cinacalcet use 1 year after transplantation. This would help clinicians determine which patients are more likely to profit from early parathyroidectomy versus medical management until spontaneous resolution.

## 2. Materials and Methods

This retrospective study was carried out in a large academic hospital in Montreal, Quebec, Canada. The study protocol was approved by our institution's research ethics committee. All patients receiving cinacalcet up to kidney transplantation and/or thereafter were included. Patients were identified using electronic health records. Data were collected from the time of transplantation up to January 1, 2013, by reviewing medical charts and laboratory results. Laboratory results were available the day of transplantation, every 3 months in the first 2 years, and every 6 months thereafter. Presumptive cause of ESRD was extracted from patients' pretransplant assessment notes. It was not always specified whether the diagnosis was biopsy proven or not.

Collected data were analyzed using commercially available statistical software. Continuous variables were expressed as mean ± standard deviation. Fisher's exact test was used to compare categorical variables and the Mann-Whitney-Wilcoxon test was used for continuous variables. Baseline variables with *p* values < 0.2 were considered in univariate and multivariate logistic regression models.

## 3. Results

**Figure 1:**
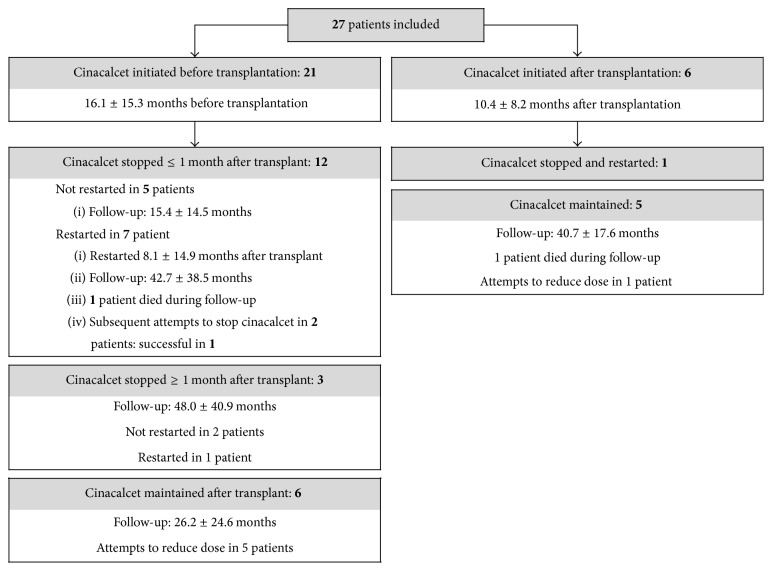
Patterns of cinacalcet use. All reported follow-up times start at kidney transplantation.

**Figure 2:**
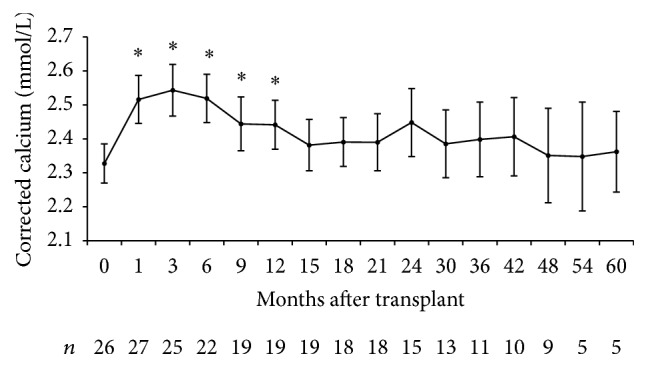
Evolution of corrected calcium after kidney transplantation. ^*∗*^*p* value < 0.05 compared to *t* = 0.

**Figure 3:**
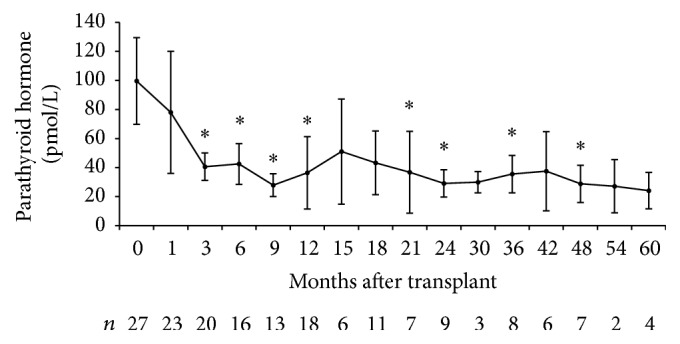
Evolution of parathyroid hormone after kidney transplantation. ^*∗*^*p* value < 0.05 compared to *t* = 0.

**Figure 4:**
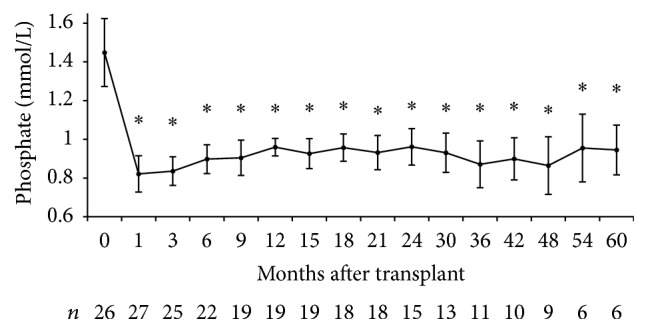
Evolution of serum phosphate after kidney transplantation. ^*∗*^*p* value < 0.05 compared to *t* = 0.

A total of 449 patients underwent kidney transplantation from March 2003 to October 2012. Baseline PTH was available in 390 patients and was 48.6 ± 45.7 pmol/L. Twenty-seven patients were included in the study consisting mainly of middle-aged Caucasian men (Table 1). Graft function was satisfactory throughout follow-up with a mean estimated glomerular filtration rate 1 year after transplantation of 49 ± 16 mL/min per 1.73 m^2^. Six patients initiated cinacalcet only after transplantation. The remaining 21 were already taking cinacalcet prior to transplantation. Cinacalcet was stopped within the first month in 12 of these patients of which 7 had to restart therapy (Figure 1). The main reason for restarting cinacalcet was hypercalcemia. Calcium levels increased after kidney transplantation especially in the first 6 months (Figure 2). On the other hand, PTH decreased considerably in the first 3 months but remained well above normal for the remainder of the follow-up (Figure 3). Baseline PTH in the study participants was 99.6 ± 79.3 pmol/L. Serum phosphate levels decreased rapidly after transplantation and remained within the normal range (Figure 4).

Cinacalcet use among all participants averaged 23.5 ± 25.6 months. There were only 3 cases of mild hypocalcemia (lowest value of 1.98 mmol/L). There were no reports of adverse events and all attempts to stop cinacalcet or reduce dose aimed to lighten the patient's “pill burden.” Average length of follow-up was 2.9 ± 2.4 years. At the end of the study, only 8 patients had stopped cinacalcet. Attempts to reduce dose or to stop cinacalcet altogether were carried out in over 80% of patients (Figure 2). One patient underwent parathyroidectomy while 5 others were awaiting operating-room availability or surgery consultation.

Among the 21 patients already taking cinacalcet, follow-up of at least 12 months was available in 17 (Table 2). Using logistic regression models, there was no statistically significant association between baseline factors and cinacalcet status at one year (Table 3).

Typical maintenance immunosuppression included prednisone, mycophenolate, and a calcineurin inhibitor (Table 4). Most patients were receiving calcium and a vitamin D analogue during the first month after transplantation. In the following months and years, these rates dropped considerably.

## 4. Discussion

These results bring to light existing challenges in the management of hyperparathyroidism in kidney transplant recipients. In the majority of patients, efforts were made to either stop cinacalcet or reduce the dose. These attempts were predominantly unsuccessful owing to recurrent hypercalcemia. Consistent with previous studies, laboratory results revealed a transient increase in calcemia within the first 3 months after transplantation. This is likely due to restored calcitriol production and PTH-induced calcium release from bone [6, 12]. Hypercalcemia is presumably the reason why the majority of these patients did not have vitamin D or calcium supplements as would be expected with prolonged corticosteroid therapy. Cinacalcet use among all participants averaged 23.5 ± 25.6 months. There were no significant adverse effects reported suggesting good tolerability. These results support cinacalcet use in a transplant setting as a bridge to parathyroidectomy or as an alternative in the case of hypercalcemia. However, it is important to reiterate that cinacalcet is not yet approved for use in renal transplant recipients. Moreover it is not clear whether either of these treatments is appropriate even if safety and biochemical efficacy are assumed [11]. Bone disease following kidney transplantation is a complex problem with multiple etiologies related to dialysis, corticosteroid therapy, calcineurin inhibitors, and persistent hyperparathyroidism [13]. Studies of bone histomorphology in renal transplant patients have shown surprisingly high prevalence of adynamic bone disease (low-bone turnover) despite hypercalcemia and hyperparathyroidism [14]. Therefore, there is a concern that cinacalcet may exacerbate low-bone turnover [15]. There is in fact a lack of published data showing improved clinical outcomes such as reduced fractures or increased bone mineral density. This raises the question of cost-effectiveness in comparison with parathyroidectomy [16] especially given prolonged survival of renal transplant recipients.

For our secondary objective, we could not identify any baseline factors predictive of cinacalcet status one year after kidney transplantation. This is certainly due to insufficient statistical power because of limited sample size.

## 5. Limitation of the Study

Inherent to the study's retrospective design, selection and information biases could not be excluded. Additionally, this was a single-center study and there was no control group for comparison. Sample size was rather small but similar to other published observational studies. However, the length of follow-up and the duration of cinacalcet use after kidney transplantation were longer than previous studies.

## 6. Conclusion

Spontaneous resolution of hyperparathyroidism after transplantation is uncommon despite lengthy follow-up and satisfactory graft function. Discontinuing cinacalcet within the first month of kidney transplantation often leads to hypercalcemia. Cinacalcet appears to be an effective treatment of persistent hyperparathyroidism and may serve as a bridge to parathyroidectomy or as an alternative. Further studies are needed to evaluate safety and long-term benefits. No baseline factors could be identified as predictors of cinacalcet use 1 year after transplantation.

## Figures and Tables

**Table 1:** Patient characteristics.

Characteristics	*n* = 27
Age (years)	52 ± 12
Male	17 (63%)
Body mass index (kg/m^2^)	26.9 ± 5.2
Ethnicity	
Caucasian	16 (59%)
Black	8 (30%)
Presumptive cause of ESRD	
Hypertensive nephrosclerosis	6 (22%)
Chronic glomerulonephritis	6 (22%)
Diabetic nephropathy	5 (19%)
Other	10 (37%)
Comorbidities	
Hypertension	26 (97%)
Dyslipidemia	20 (74%)
Diabetes	7 (26%)
Coronary artery disease	9 (33%)
Smoking	5 (19%)
Dialysis modality	
In-center hemodialysis	17 (63%)
Home hemodialysis	4 (15%)
Peritoneal dialysis	5 (19%)
Predialysis	1 (4%)
Time spent on dialysis (years)	5.8 ± 4.3
Prior kidney transplantation	3 (11%)
Prior subtotal parathyroidectomy	2 (7%)
Follow-up after transplant (years)	2.9 ± 2.4
Cinacalcet use after transplant (months)	23.5 ± 25.6

Continuous variables are expressed as mean ± standard deviation; categorical variables are expressed as count (%).

**Table 2:** Baseline characteristics according to cinacalcet status 1 year after kidney transplantation.

Characteristics	No cinacalcet *n* = 6	Cinacalcet *n* = 11	*p* value
Age (years)	51 ± 11	55 ± 10	ns
Male	4 (67%)	9 (82%)	ns
Body mass index (kg/m^2^)	23.4 ± 4.9	27.1 ± 4.0	0.105
Ethnicity			
Caucasian	3 (50%)	6 (55%)	ns
Black	2 (33%)	4 (36%)	ns
Presumptive cause of ESRD			
Diabetic nephropathy	1 (17%)	3 (27%)	ns
Hypertensive nephrosclerosis	2 (33%)	3 (27%)	ns
Polycystic renal disease	1 (17%)	3 (27%)	ns
Other	2 (33%)	2 (18%)	ns
Comorbidities			
Hypertension	6 (100%)	11 (100%)	ns
Dyslipidemia	4 (67%)	9 (82%)	ns
Diabetes	1 (17%)	4 (36%)	ns
Coronary artery disease	2 (33%)	4 (36%)	ns
Heart failure	0 (0%)	6 (55%)	0.043
Smoking	1 (17%)	1 (9%)	ns
Dialysis modality			
In-center hemodialysis	4 (67%)	6 (55%)	ns
Home hemodialysis	1 (17%)	3 (27%)	ns
Peritoneal dialysis	1 (17%)	2 (18%)	ns
Time spent on dialysis (years)	8.7 ± 5.0	6.9 ± 3.8	ns
Baseline biochemistry			
PTH (pmol/L)	142.5 ± 140.2	66.7 ± 35.0	0.102
Corrected calcium (mmol/L)	2.3 ± 0.1	2.3 ± 0.1	ns
Phosphate (mmol/L)	1.6 ± 0.6	1.2 ± 0.3	0.196

PTH: parathyroid hormone; ns: not significant; *p* values < 0.05 are considered statistically significant; *p* values < 0.2 are indicated because variables were included in logistic regression model; continuous variables are expressed as mean ± standard deviation; categorical variables are expresses as count (%).

**Table 3:** Logistic regression models for cinacalcet status 1 year after kidney transplantation.

Variables	Unit change	Univariate model		Multivariate model	
		OR	95% CI	OR	95% CI
BMI (kg/m^2^)	5	2.79	0.79; 9.82	1.97	0.52; 7.52
Phosphate (mmol/L)	0.1	0.80	0.60; 1.08	0.92	0.74; 1.13
PTH (pmol/L)	10	0.89	0.76; 1.05	1.04	0.69; 1.56
Heart failure	1 versus 0	15.37	0.55; 427.5	9.52	0.39; 231.3

BMI: body mass index; PTH: parathyroid hormone; only variables with *p* values < 0.2 were included in logistic regression models; the odds ratio (OR) of having cinacalcet 1 year after kidney transplantation is reported for a determined unit change of each variable using univariate and multivariate models. Each odds ratio is reported with a 95% confidence interval (95% CI).

**Table 4:** Medication after kidney transplantation.

Time after transplantation	0	1 month	12 months	24 months	36 months
Available data	27	27	19	15	11
Calcium carbonate	19 (70%)	13 (48%)	4 (21%)	2 (13%)	0 (0%)
Vitamin D analogues	21 (78%)	18 (67%)	7 (37%)	6 (40%)	4 (36%)
Cholecalciferol	1 (4%)	0 (0%)	1 (5%)	0 (0%)	1 (9%)
Alfacalcidol	13 (48%)	11 (41%)	3 (16%)	4 (27%)	2 (18%)
Calcitriol	8 (30%)	7 (26%)	3 (16%)	2 (13%)	2 (18%)
Bisphosphonate		16 (59%)	12 (63%)	10 (67%)	8 (73%)
Immunosuppression					
Prednisone		27 (100%)	19 (100%)	15 (100%)	11 (100%)
CNI		25 (93%)	17 (89%)	12 (80%)	8 (73%)
Mycophenolate		27 (100%)	16 (84%)	13 (87%)	9 (82%)
Azathioprine		0 (0%)	1 (5%)	2 (13%)	2 (18%)
mTOR inhibitor		2 (7%)	2 (11%)	3 (20%)	3 (27%)

CNI: calcineurin inhibitors; mTOR: mammalian target of rapamycin; results are expressed as count (% of the available data at that point in time).
